# Colibactin-Producing *Escherichia coli* Induce the Formation of Invasive Carcinomas in a Chronic Inflammation-Associated Mouse Model

**DOI:** 10.3390/cancers13092060

**Published:** 2021-04-24

**Authors:** Laurène Salesse, Cécily Lucas, My Hanh Thi Hoang, Pierre Sauvanet, Alexandra Rezard, Philip Rosenstiel, Christelle Damon-Soubeyrand, Nicolas Barnich, Catherine Godfraind, Guillaume Dalmasso, Hang Thi Thu Nguyen

**Affiliations:** 1 M2iSH, UMR 1071 Inserm, Université Clermont Auvergne, INRAE USC 2018, CRNH, 63001 Clermont-Ferrand, France; laurene.salesse@uca.fr (L.S.); cecily.lucas@uca.fr (C.L.); thi_my_hanh.hoang@uca.fr (M.H.T.H.); pierre.sauvanet@uca.fr (P.S.); alexandra.rezard@uca.fr (A.R.); nicolas.barnich@uca.fr (N.B.); cgodfraind@chu-clermontferrand.fr (C.G.); guillaume.dalmasso@uca.fr (G.D.); 2 Department of Cell Biology, Faculty of Biology, University of Science, Vietnam National University (VNU), Hanoi 100000, Vietnam; 3 Department of Digestive and Hepatobiliary Surgery, CHU Estaing, 63001 Clermont-Ferrand, France; 4 Institute of Clinical Molecular Biology, Christian-Albrechts-University and University Hospital Schleswig-Holstein, 24148 Kiel, Germany; p.rosenstiel@mucosa.de; 5 GReD, CNRS UMR 6293 INSERM U110, Université Clermont Auvergne, 63001 Clermont-Ferrand, France; christelle.soubeyrand-damon@uca.fr; 6 Department of Pathology, CHU Gabriel Montpied, 63001 Clermont-Ferrand, France

**Keywords:** colorectal cancer, microbiota, colibactin-producing *E. coli*, autophagy, toxin

## Abstract

**Simple Summary:**

Changes in the composition of the intestinal flora have been reported in patients with colorectal cancer, the second leading cause of cancer death in the world, with an increase in so-called "harmful" bacteria. Among these, *Escherichia coli* producing colibactin, a toxin that causes DNA damage, has attracted the interest of many research groups. Here, we showed that infection of wild-type mice with a colibactin-producing *E. coli* (CoPEC) strain, isolated from a patient with colorectal cancer, combined with chronic inflammation induced the formation of invasive colonic tumors, i.e., tumors that spread beyond epithelial layer and grow into surrounding tissues. We also showed that autophagy, a cell defense process, is necessary to inhibit the tumorigenesis induced by CoPEC. Thus, this work highlights the role of CoPEC as a driver of colorectal cancer development, and suggests that targeting autophagy could be a promising strategy to inhibit the protumoral effects of these bacteria.

**Abstract:**

Background: *Escherichia coli* producing the genotoxin colibactin (CoPEC or colibactin-producing *E. coli*) abnormally colonize the colonic mucosa of colorectal cancer (CRC) patients. We previously showed that deficiency of autophagy in intestinal epithelial cells (IECs) enhances CoPEC-induced colorectal carcinogenesis in *Apc^Min/+^* mice. Here, we tested if CoPEC trigger tumorigenesis in a mouse model lacking genetic susceptibility or the use of carcinogen. Methods: Mice with autophagy deficiency in IECs (*Atg16l1*^∆*IEC*^) or wild-type mice (*Atg16l1^flox/flox^*) were infected with the CoPEC 11G5 strain or the mutant 11G5∆clbQ incapable of producing colibactin and subjected to 12 cycles of DSS treatment to induce chronic colitis. Mouse colons were used for histological assessment, immunohistochemical and immunoblot analyses for DNA damage marker. *Results*: 11G5 or 11G5∆clbQ infection increased clinical and histological inflammation scores, and these were further enhanced by IEC-specific autophagy deficiency. 11G5 infection, but not 11G5∆clbQ infection, triggered the formation of invasive carcinomas, and this was further increased by autophagy deficiency. The increase in invasive carcinomas was correlated with enhanced DNA damage and independent of inflammation. *Conclusions*: CoPEC induce colorectal carcinogenesis in a CRC mouse model lacking genetic susceptibility and carcinogen. This work highlights the role of (i) CoPEC as a driver of CRC development, and (ii) autophagy in inhibiting the carcinogenic properties of CoPEC.

## 1. Introduction

Colorectal cancer (CRC) is the third most common cancer around the world, with 1.85 million of new cases per year and the second leading cause of death by cancer with 880,000 recorded deaths worldwide [1]. The etiology of CRC is multifactorial, including age, genetics, lifestyle, diet, and environmental factors [2]. An association between chronic intestinal inflammation, as observed in inflammatory bowel disease, and an increased risk for CRC development has been reported and called by colitis-associated CRC [3].

The involvement of gut microbiota in CRC pathogenesis has received much attention during the past few years [2]. Metagenomic approaches have shown that in CRC patients, compared to healthy subjects, the gut microbiota has lower abundance of protective taxa and higher abundance of procarcinogenic taxa such as *Bacteroides*, *Escherichia* and *Fusobacterium* [4,5]. To support a role for the gut microbiota in colorectal carcinogenesis, studies using carcinogen-induced or genetically susceptible mouse models have been performed. One of the most common genetically susceptible mouse models of CRC is *Apc^Min/+^* mice, which carry a loss-of-function germinal mutation in the *Apc* gene, leading to spontaneous formation of multiple adenomas in the intestine and the colon [6]. *Apc^Min/+^* mice bearing microbiota develop more intestinal and colorectal tumors compared with germ-free *Apc^Min/+^* mice [7]. Recently, it was shown that *Apc^Min/+^* mice receiving the fecal samples from CRC patients develop increased number of intestinal adenomas and a more advanced tumor development compared with those receiving fecal samples from healthy subjects [8]. In a colitis-associated CRC mouse model chemically induced by azoxymethane (AOM) and dextran sodium sulfate (DSS), antibiotic treatment significantly decreases the number, the size and the histological score of the colonic tumors [9]. Furthermore, transfer of fecal samples from patients with CRC enhances intestinal cell proliferation in germ-free mice and promotes tumor formation in conventional mice that were pre-treated with antibiotics and then with AOM [10].

Besides these findings showing a direct association between the gut microbiota and CRC development, increasing evidence has revealed the effects of bacteria-derived products on carcinogenesis. Among these, the carcinogenic compound colibactin, a polyketide-derived genotoxin produced by *Escherichia coli*, has attracted interest from researchers. Colibactin-producing *Escherichia coli* (CoPEC) have been shown to be more prevalent in the biopsies of CRC patients compared to control patients [11,12,13,14]. CoPEC are more frequently identified in CRC patients with tumor-node-metastasis (TNM) stage II and III/IV than in patients with TNM stage I, suggesting an association between CoPEC colonization and the poor prognostic factors for CRC [15]. In vitro and in vivo studies have demonstrated the carcinogenic properties of colibactin and the implication of CoPEC in colorectal carcinogenesis [2]. Colibactin induces DNA interstrand cross-links, that is converted into DNA double-strand breaks (DSBs) in mammalian cells during the repair response, leading to chromosomal aberrations and cell cycle arrest [16,17,18]. A recent study showed that colibactin alkylates DNA in vivo, generating DNA adducts in mammalian cells and in germ-free mice [19]. Importantly, a direct link between a distinct mutational signature caused by exposure of human intestinal epithelial cells (IECs) to CoPEC and known CRC driver mutations was shown [20].

Infection with CoPEC has been shown to promote colon tumorigenesis in mouse models of CRC, such as *Apc^Min/+^* mice [15,21], AOM-treated *il10^−/−^* mice [11], AOM/DSS-treated mice [22] or *Apc^Min/+^*/*il10^−/−^* mice [23]. Different mechanisms underlying the carcinogenic effects of CoPEC infection have been revealed. For example, following the formation of DNA DSBs, the cells infected with CoPEC undergo cell cycle arrest and display cellular senescence [22]. This phenotype is accompanied by the production of inflammatory mediators and growth factors, promoting the proliferation of uninfected cells and tumorigenesis in CRC predisposed mice [22]. We recently showed that autophagy, a highly regulated stress responsive process that degrades potentially dangerous cytosolic components via the lysosomal pathway [24], is a key mechanism of host defense to CoPEC infection [21]. Indeed, autophagy in IECs is necessary to inhibit the genotoxic and protumoral properties of CoPEC, limiting CoPEC-induced colorectal carcinogenesis in *Apc^Min/+^* mice [21].

So far, investigations of the protumoral effects of CoPEC have been performed in genetically susceptible mouse models of CRC, such as *Apc^Min/+^* mice [15,21], or mouse models including the use of a carcinogen [11,22]. In the current study, we tested the ability of CoPEC to induce tumorigenesis in a mouse model with DSS-induced chronic inflammation which lacks genetic susceptibility or the use of a carcinogen, and examined the impact of IEC-specific autophagy deficiency on tumorigenesis in these models.

## 2. Results

### 2.1. CoPEC Infection Increases Clinical Score in a Mouse Model of Chronic DSS Treatment, Independently of Colibactin, and This Is Further Enhanced by Intestinal Autophagy Deficiency

**Figure 1:**
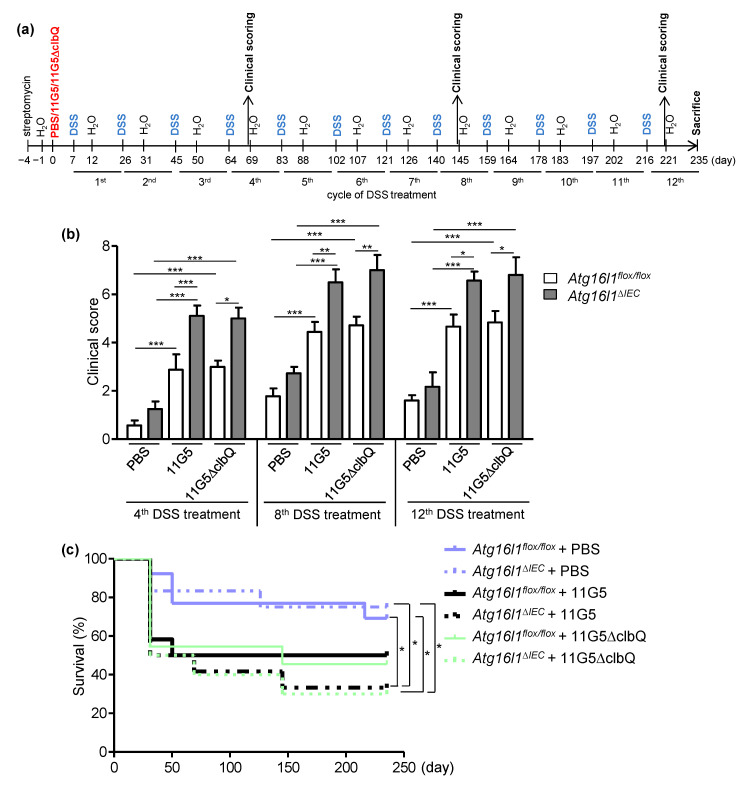
CoPEC infection increases the susceptibility of mice to the chronic DSS treatment independently of colibactin, and this is further enhanced by autophagy deficiency in IECs. (**a**) In vivo infection protocol. *Atg16l1^flox/flox^* and *Atg16l1^∆IEC^* mice were treated with streptomycin for 3 days and then received water for 24 h before being orally administered (day 0) with PBS or with 10^9^ colony-forming units of 11G5 bacteria or the mutant 11G5∆clbQ. Seven days later, the mice were subjected to 12 cycles of DSS treatment, with each cycle consisted of 1% of DSS in drinking water for 5 days followed by 14-day of regular water. (**b**) Clinical activity score was determined based on the assessment of body weight loss, stool consistency, and the presence of occult blood in the stool after the 4th, 8th and 12th DSS treatment (after 5 days of treatment, just before the mice were given regular water). Results are means ± SEM. *N* = 10 mice/group for + PBS and 11G5-infected conditions; *N* = 6 mice/group for 11G5∆clbQ-infected condition. (**c**) Survival of mice during the chronic DSS treatment. *Atg16l1^flox/flox^* + PBS: *N* = 13; *Atg16l1^∆IEC^* + PBS: *N* = 12; *Atg16l1^flox/flox^* + 11G5: *N* = 12; *Atg16l1^∆IEC^* + 11G5: *N* = 12; *Atg16l1^flox/flox^* + 11G5∆clbQ: *N* = 11; *Atg16l1^∆IEC^* + 11G5∆clbQ: *N* = 10. * *p* < 0.05; ** *p* ≤ 0.01; *** *p* ≤ 0.001 by one-way Anova, followed by a post-test Bonferroni in (**b**) and Gehan-Breslow-Wilcoxon test in (**c**).

Previous studies have shown that CoPEC infection promotes colon tumorigenesis in genetically susceptible or carcinogen-induced mouse models of CRC [2]. Here, we investigated whether CoPEC infection combined with chronic inflammation can enhance colon tumorigenesis in wild-type mice. The role of autophagy in this model was also investigated as we previously showed that autophagy in IECs is necessary to inhibit CoPEC-induced colon tumorigenesis in *Apc^Min/+^* mice [21]. For this, mice deficient for the autophagy-related gene *Atg16l1* specifically in IECs (*Atg16l1^flox/flox^CreVillin* or *Atg16l1^∆IEC^*) or the wild-type mice (*Atg16l1^flox/flox^*) were infected with the CoPEC 11G5 strain isolated from a CRC patient or a mutant strain that does not produce colibactin (11G5∆clbQ) and were subjected to 12 cycles of DSS treatment (each cycle consisted of 1% of DSS in drinking water for 5 days followed by 14-day of regular water; Figure 1a).

Under 11G5 or 11G5∆clbQ infection, *Atg16l1^flox/flox^* or *Atg16l1^∆IEC^* mice displayed a higher susceptibility to DSS treatment, compared to uninfected mice (or + PBS groups, i.e., the mice were gavaged only with PBS), as shown by higher clinical disease score, determined at the end of each DSS treatment based on 3 parameters, including body weight loss, stool consistency, and the presence of occult blood in the stool (Figure 1b). No significant difference in clinical disease score to the chronic DSS treatment was observed between 11G5-infected and 11G5∆clbQ-infected groups (Figure 1b). Compared to *Atg16l1^flox/flox^* mice, *Atg16l1^∆IEC^* mice exhibited increased clinical disease score upon DSS treatment under both 11G5- or 11G5∆clbQ-infected conditions (Figure 1b). We noticed that the clinical score of all groups tended to be increased after the 8th DSS treatment compared to the 4th DSS treatment, although the difference was not significant (Figure 1b). Furthermore, during the chronic DSS treatment, a significant increase in mortality was observed for 11G5- or 11G5∆clbQ-infected *Atg16l1^∆IEC^* mice compared to + PBS groups (Figure 1c). Together, these data showed that CoPEC infection increases the susceptibility of mice to the chronic DSS treatment, independently of colibactin, and this was further enhanced by autophagy deficiency in IECs. Combination of autophagy deficiency in IECs and CoPEC infection enhances the mortality of mice compared to uninfected condition.

### 2.2. CoPEC Infection Promotes Colon Tumorigenesis in a Mouse Model of Chronic DSS Treatment, and This Was Further Enhanced by Autophagy Deficiency in IECs

**Figure 2:**
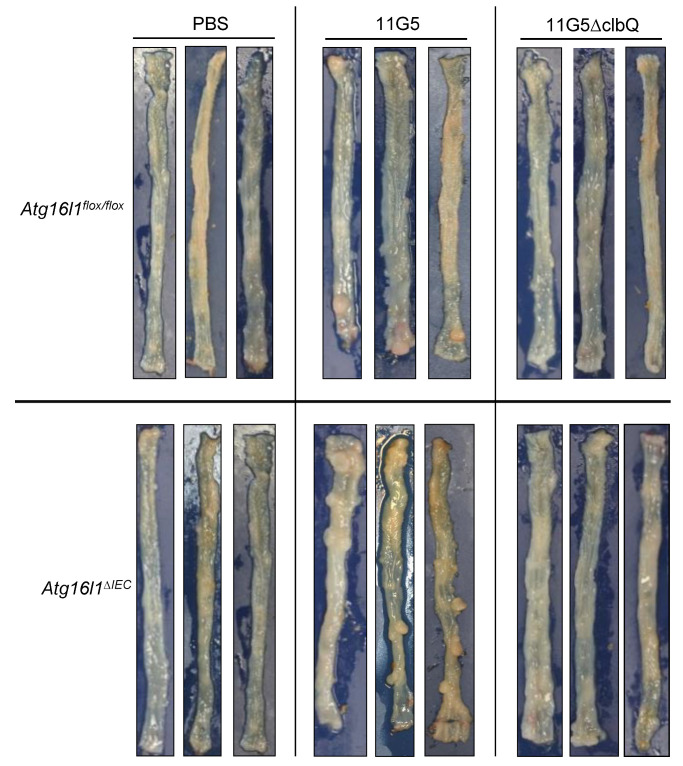
CoPEC infection induces colon tumorigenesis in a mouse model of chronic DSS treatment, and this is further enhanced by autophagy deficiency in IECs. *Atg16l1^flox/flox^* and *Atg16l1^∆IEC^* mice were treated with streptomycin for 3 days and then received water for 24 h before being orally administered (day 0) with PBS or with 10^9^ colony-forming units of 11G5 bacteria or the mutant 11G5∆clbQ. Seven days later, the mice were subjected to 12 cycles of DSS treatment. Representative photos of the colons taken at the sacrifice were shown. The images are representatives of *N* = 10 mice/group for +PBS and 11G5-infected conditions, and *N* = 6 mice/group for 11G5∆clbQ-infected condition.

**Figure 3:**
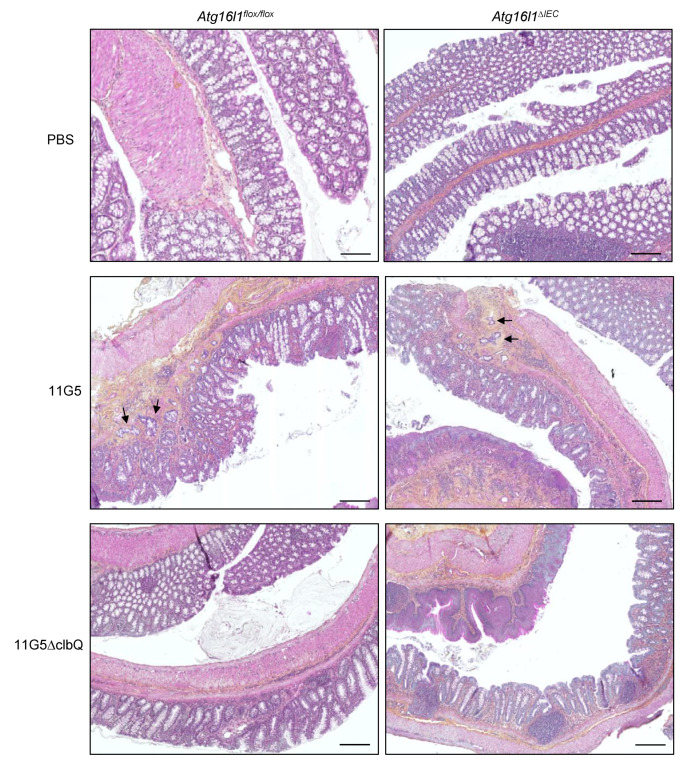
CoPEC infection triggers the formation of invasive adenocarcinomas in a mouse model of chronic DSS treatment, and this was further enhanced by autophagy deficiency in IECs. *Atg16l1^flox/flox^* and *Atg16l1^∆IEC^* mice were treated with streptomycin for 3 days and then received water for 24 h before being orally administered (day 0) with PBS or with 10^9^ colony-forming units of 11G5 bacteria or the mutant 11G5∆clbQ. Seven days later, the mice were subjected to 12 cycles of DSS treatment. Sections of the colons taken at the sacrifice were H&E-stained. The images are representatives of *N* = 10 mice/group for +PBS and 11G5-infected conditions, and *N* = 6 mice/group for 11G5∆clbQ-infected condition. Black arrows show invasion of the glands through the muscularis mucosa. Bars = 200 µm.

**Figure 4:**
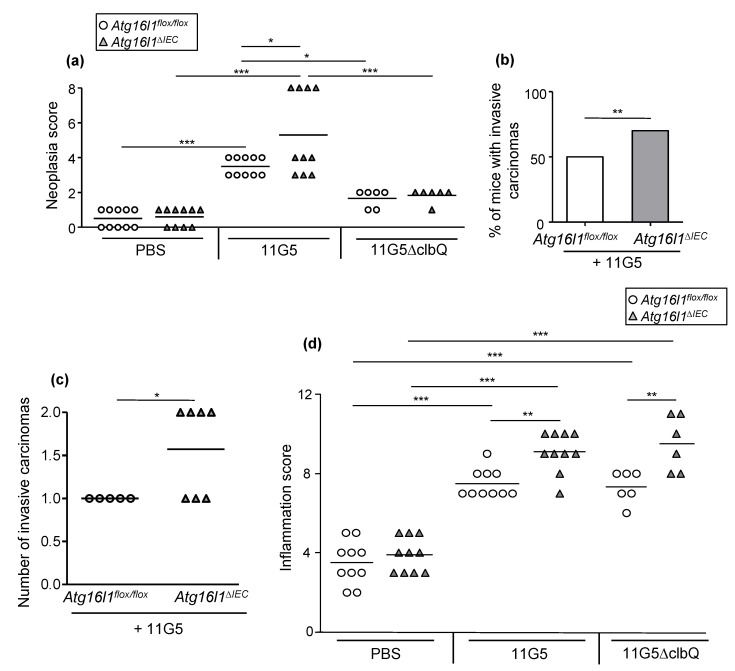
CoPEC infection induces colon tumorigenesis in a mouse model of CRC, independently of inflammation, and this was further enhanced by intestinal autophagy deficiency. *Atg16l1^flox/flox^* and *Atg16l1^∆IEC^* mice were treated with streptomycin for 3 days and then received water for 24 h before being orally administered (day 0) with PBS or with 10^9^ colony-forming units of 11G5 bacteria or the mutant 11G5∆clbQ. Neoplasia score (**a**), percentage of mice with invasive adenocarcinomas (**b**), number of invasive carcinomas (**c**) and inflammation score (**d**) were determined based on the histological evaluation of the colonic sections under blinded conditions by an expert pathologist. * *p* < 0.05; ** *p* ≤ 0.01; *** *p* ≤ 0.001 by one-way Anova, followed by a post-test Bonferroni in (**a**,**d**), the Fisher exact test in (**b**), and two-way *t* test in (**c**). (**a**,**c**,**d**) Each symbol represents data from one mouse, lines at mean. (**a**,**b**,**d**) *N* = 10 mice/group for +PBS and 11G5-infected conditions, and *N* = 6 mice/group for 11G5∆clbQ-infected condition. (**c**) *Atg16l1^flox/flox^* + 11G5: *N* = 5; *Atg16l1^∆IEC^* + 11G5: *N* = 7.

Importantly, infection with 11G5, but not the mutant 11G5∆clbQ, triggered the formation of adenocarcinomas as shown by representative images of the colons of mice at sacrifice (Figure 2) and histological examination (Figure 3). Histological examination revealed invasive carcinomas in 11G5-infected groups, but not in +PBS or 11G5∆clbQ-infected groups (Figure 3). Invasion of the glands through the muscularis mucosa into the submucosa was observed in both 11G5-infected *Atg16l1^flox/flox^* and 11G5-infected *Atg16l1^∆IEC^* mice (black arrows, Figure 3). Infection with 11G5 was associated with significantly increased neoplasia score, determined based on the criteria in Table 1, compared to +PBS and 11G5∆clbQ-infected conditions for both *Atg16l1^flox/flox^* and *Atg16l1^∆IEC^* mice (Figure 3 and Figure 4a).

Autophagy deficiency in IECs further enhanced neoplasia score in 11G5-infected mice, but not in 11G5∆clbQ-infected mice (Figure 3 and Figure 4a). Furthermore, 70% of 11G5-infected *Atg16l1^∆IEC^* mice surviving after the chronic DSS treatment developed carcinomas in the colon, whereas 50% of 11G5-infected *Atg16l1^flox/flox^* mice developed these (** *p* = 0.0059; Figure 4b). The number of invasive carcinomas in 11G5-infected *Atg16l1^∆IEC^* mice (1.571 ± 2.202) was also increased compared to that in 11G5-infected *Atg16l1^flox/flox^* mice (1 ± 0) (* *p* = 0.04; Figure 4c).

Together, these data indicated that CoPEC infection induces colon tumorigenesis in a mouse model of chronic DSS treatment. Deficiency of autophagy in IECs led to increased number of 11G5-infected mice to develop invasive carcinomas and increased number of invasive carcinomas.

### 2.3. CoPEC Infection and Autophagy Deficiency in IECs Enhance Colonic Inflammation in Chronic DSS Treatment Independently of Colibactin

As inflammation is an important factor contributing to CRC development, we next examined intestinal inflammation in *Atg16l1^flox/flox^* and *Atg16l1^∆IEC^* mice. Histological examination showed that infection with 11G5 or 11G5∆clbQ was associated with enhanced histological inflammation score, determined based on the criteria in Table 2 (Figure 4d). Intestinal autophagy deficiency aggravated inflammation score in both 11G5-infected and 11G5∆clbQ-infected mice, but not in + PBS mice (Figure 4d). No difference in inflammation score were found between 11G5-infected and 11G5∆clbQ-infected groups (Figure 4d).

Together, these data indicated that CoPEC infection and autophagy deficiency in IECs enhance colonic inflammation independently of colibactin.

### 2.4. Autophagy Is Necessary to Limit CoPEC-Induced Colonic DNA Damage in the Mouse Model of Chronic DSS Treatment

**Figure 5:**
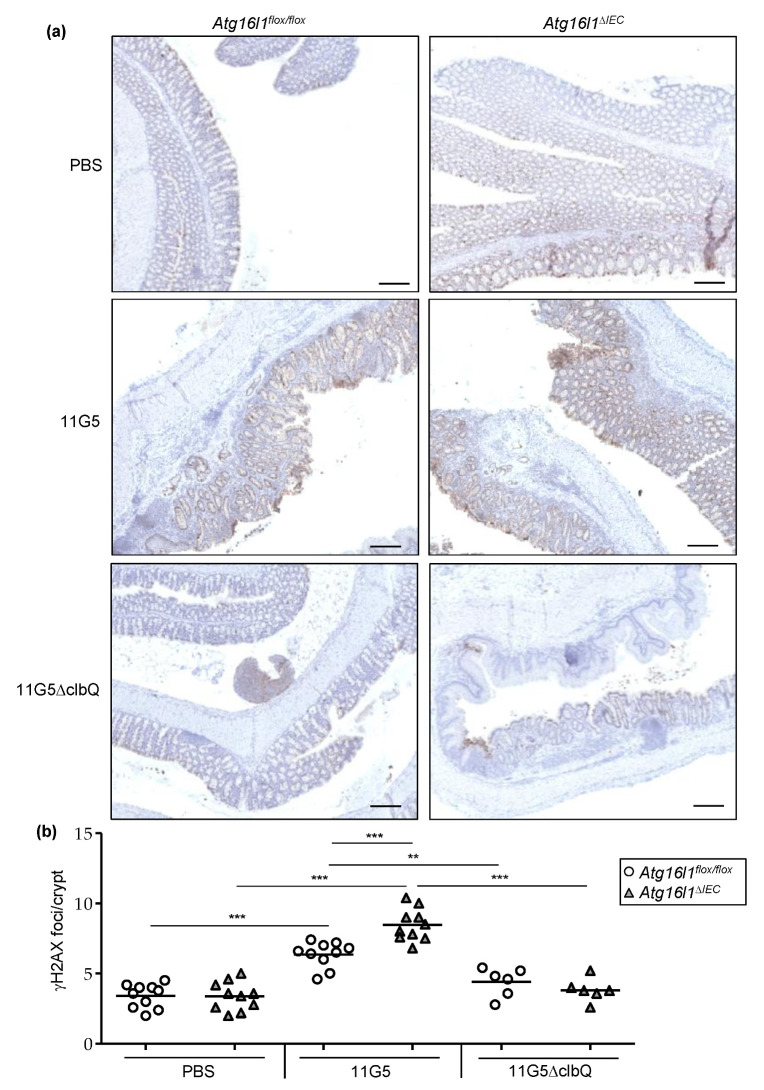
CoPEC infection increases marker of DNA damage in colonic crypt in a mouse model of chronic DSS treatment, and this was further enhanced by autophagy deficiency in IECs. *Atg16l1^flox/flox^* and *Atg16l1^∆IEC^* mice were treated with streptomycin for 3 days and then received water for 24 h before being orally administered (day 0) with PBS or with 10^9^ colony-forming units of 11G5 bacteria or the mutant 11G5∆clbQ. (**a**) Immunohistochemical staining for γH2AX in mouse colonic sections. Bars = 200 µm. (**b**) Quantification of γH2AX foci number per crypt determined from 20 crypts/mouse, *N* = 10 mice/group for +PBS or 11G5-infected mice, *N* = 6 mice/group for 11G5∆clbQ-infected mice. Each symbol represents data from one mouse, lines at mean. ** *p* ≤ 0.01; *** *p* ≤ 0.001 by one-way ANOVA, followed by a post-test Bonferroni.

We next investigated the role of colibactin and autophagy in CoPEC-induced DNA damage in the chronic DSS treatment. Quantification of H2AX phosphorylation (γH2AX) foci as well as foci formed by other repair proteins, such as MRE11, 53BP1, RAD51, etc., has been used to detect DNA DSBs [25]. Here, we performed immunohistochemical staining to detect γH2AX as this marker has been used in numerous important studies to show CoPEC-induced DNA damage [11,17,18,21,22]. We showed that 11G5-infected *Atg16l1^flox/flox^* mice exhibited increased number of γH2AX foci per colonic crypt compared to *Atg16l1^flox/flox^* + PBS or 11G5∆clbQ-infected *Atg16l1^flox/flox^* mice (Figure 5a,b). Importantly, the number of γH2AX foci per colonic crypt was higher in 11G5-infected *Atg16l1^∆IEC^* mice compared to 11G5-infected *Atg16l1^flox/flox^* mice (Figure 5a,b).

**Figure 6:**
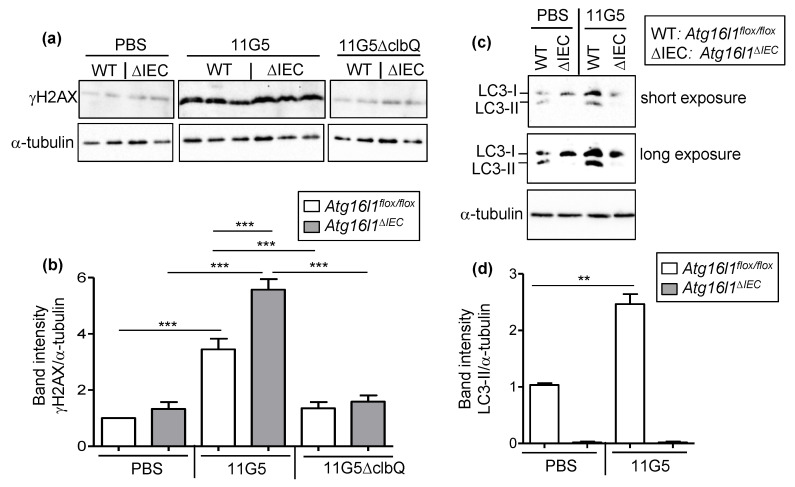
CoPEC infection increases the level of DNA damage marker in the colons of chronic DSS-treated mice, and this was further enhanced by intestinal autophagy deficiency. *Atg16l1^flox/flox^* (WT) and *Atg16l1^∆IEC^* (∆IEC) mice were treated with streptomycin for 3 days and then received water for 24 h before being orally administered (day 0) with PBS or with 10^9^ colony-forming units of 11G5 bacteria or the mutant 11G5∆clbQ. (**a**,**c**) Representative western blot analysis of γH2AX (**a**) and LC3 (**c**) levels in the colonic mucosa. The uncropped Western blots have been shown in Figure S1. (**b**,**d**) Quantification of western blot band intensity from *N* = 6/group. Results are means ± SEM. ** *p* ≤ 0.01; *** *p* ≤ 0.001 by one-way ANOVA, followed by a post-test Bonferroni.

These results were further confirmed by western blot analysis, which showed increased level of γH2AX in the colonic mucosa of 11G5-infected *Atg16l1^flox/flox^* mice compared to *Atg16l1^flox/flox^* + PBS or 11G5∆clbQ-infected *Atg16l1^flox/flox^* mice (Figure 6a,b). Upon 11G5 infection, there was an increase in γH2AX level in the colonic mucosa of *Atg16l1^∆IEC^* compared to *Atg16l1^flox/flox^* mice (Figure 6a,b). Finally, autophagy status in the colonic mucosa from these mice was verified by western blot analysis for the levels of LC3-I (cytosolic form) and LC3-II (autophagosome-associated form and a marker of autophagy [26]) using the same lysates. As shown in Figure 6c,d, 11G5 infection resulted in increased LC3-II level, indicating autophagy activation, in the colonic mucosa of *Atg16l1^flox/flox^* mice. This was consistent with our previous study showing the activation of autophagy in IECs upon infection with the CoPEC strains [21]. As expected, we did not detect LC3-II in *Atg16l1^∆IEC^* mice under both +PBS and 11G5-infected conditions (Figure 6c,d).

Together, these data indicated that CoPEC induce colonic DNA damage in the chronic DSS treatment via producing colibactin. Intestinal autophagy is essential to limit colibactin-induce DNA damage in this mouse model.

## 3. Discussion

Mutations in core autophagy-related genes are associated with numerous diseases, such as neurological disorders, inflammatory diseases or cancers [27]. Autophagy is well known to contribute to cancer development, and autophagy gene signature is frequently associated with patient prognosis [28,29,30,31,32]. Nevertheless, the mechanism underlying the role of autophagy in colorectal carcinogenesis is still unclear as autophagy can have both pro- and anti-tumoral functions. In this regard, we previously showed the complex role of autophagy in the colorectal carcinogenesis in *Apc^Min/+^* mice under uninfected and CoPEC-infected conditions [21]. Indeed, in *Apc^Min/+^* mice, under uninfected condition, autophagy in IECs is necessary to promote colonic tumorigenesis, but upon CoPEC infection, this process limits CoPEC-induced tumorigenesis [21].

Concerning the CRC, no direct association between a specific bacterium and the cancer initiation has been described. Nevertheless, the role of autophagy in host defense against *Fusobacterium nucleatum*, a bacterium associated with colorectal carcinogenesis [2], has been investigated. It was shown that *F. nucleatum*, by targeting the TLR4-MYD88 innate immune signaling and specific microRNAs, activates autophagy to promote colorectal cancer resistance to chemotherapy [33]. Level of BECLIN 1, a key positive regulator of autophagy that initiates autophagosome formation, was recently shown to be inversely correlated with the quantity of *F. nucleatum* in colorectal tumors [34]. These findings suggest a role for autophagy in the elimination of *F. nucleatum* from the tumor microenvironment. Concerning CoPEC, which have shown great interest in CRC research [2], we recently showed that autophagy in IECs is necessary to inhibit the protumoral effects of CoPEC, suppressing colorectal carcinogenesis in CoPEC-infected *Apc^Min/+^* mice [21]. So far, the protumoral effects of CoPEC have been shown in mouse models of CRC, which present either genetic susceptibility or the use of a carcinogen [11,15,21,22,23].

Here, we demonstrated, for the first time, a link between autophagy, CoPEC and CRC by using a chronic inflammation-associated mouse model. For this, a mouse model of long-term, chronic DSS treatment was employed. It has previously been shown that CoPEC infection does not induce tumorigenesis in wild-type and untreated mice [2]. The use of DSS has been usually used following a prior administration of the carcinogen AOM to induce colorectal tumorigenesis in mice, leading to the well-established AOM/DSS model of colitis-associated CRC [35]. In this study, to better define the implication of CoPEC in carcinogenesis, the use of AOM was replaced by CoPEC infection. We showed that CoPEC infection triggers the formation of invasive adenocarcinomas, and colibactin is required for this effect. IEC-specific autophagy deficiency leads to enhanced tumorigenesis induced by CoPEC infection in this model. Importantly, we showed that the induction of tumorigenesis in the mouse model of chronic DSS treatment by CoPEC infection and intestinal autophagy deficiency was associated with increased DNA damage in the colonic crypts. We previously showed that autophagy in IECs is necessary for the recruitment of the DNA repair protein, RAD51, to the sites of DNA DSBs, limiting colibactin-induced DNA damage [21]. Thus, autophagy deficiency in IECs may lead to the appearance of mutations in the genes implicated in carcinogenesis, enhancing the formation of adenomatous lesions. Our hypothesis is strongly supported by a recent publication showing a direct role of CoPEC in the occurrence of oncogenic mutations [20]. Indeed, the whole-genome sequencing of human intestinal organoids exposed to genotoxic CoPEC revealed a distinct mutational signature that was identical with the mutational signature detected in a subset of 5,876 human cancer genomes from two independent cohorts [20].

As a close relationship between inflammation and cancer has been shown, we then investigated whether inflammation is implicated in the induction of colon tumorigenesis by CoPEC infection and autophagy deficiency in IECs. Indeed, autophagy in IECs has been shown to inhibit intestinal inflammation [36]. Another study showed that mutant mice that inducibly express UVRAG, a tumor suppressor gene involved in autophagy process, display increased inflammatory response in colitis-associated cancer development and promote spontaneous tumorigenesis associated to age-related autophagy suppression [37]. Furthermore, *H. pylori* disrupts the autophagy pathway, promoting chronic inflammation and associated damages which favor *H. pylor*i-mediated gastric tumorigenesis [38]. These results are in agreement with our previous study showing that autophagy deficiency in IECs enhances colon tumorigenesis in *Apc^M^*^in/+^ mice infected with the CoPEC 11G5 strain, and this was associated with increased mRNA levels of pro-inflammatory cytokines and chemokines in the nontumoral colonic mucosa [21]. In the current study, we showed that compared to the uninfected condition, 11G5 or 11G5∆clbQ infection increased clinical and histological inflammation scores. Furthermore, autophagy deficiency in IECs further enhanced clinical and histological inflammation scores in 11G5- or 11G5∆clbQ-infected mice, but not in uninfected mice. This was not consistent with a previous study showing that autophagy deficiency in IECs leads to increased survival, clinical disease score and colon histopathology score in mice treated with 5% DSS for 6 days under uninfected condition [39]. However, this difference could be due to the difference in DSS concentration and the time of DSS treatment. No difference in clinical and histological inflammation score was observed between 11G5- and 11G5ΔclbQ-infected conditions either in wild-type mice or mice with IEC-specific autophagy deficiency. This data was in agreement with other studies showing that CoPEC induce inflammation independently of colibactin [11,22]. Thus, these data showed that CoPEC infection and autophagy deficiency in IECs enhance colon tumorigenesis in the mouse model of chronic DSS treatment via inducing DNA damage and independently of inflammation.

In conclusion, our study showed that CoPEC exhibit their carcinogenic properties and induce the formation of invasive carcinomas in a mouse model that lacks genetic susceptibility and the use of a carcinogen. Thus, we propose that CoPEC infection combined with chronic DSS treatment could be used as a novel mouse model of CRC. We also investigated the mechanism by which autophagy in IECs inhibits CoPEC-induced colonic tumorigenesis in this mouse model, which is mediated via the inhibition of DNA damage in colonic crypts. Thus, our study importantly highlights the role of CoPEC in tumor initiation and how the host can counteract the oncogenic CoPEC through the activation of the autophagy machinery.

## 4. Materials and Methods

### 4.1. Bacterial Strains and Culture

The clinical CoPEC 11G5 strain isolated from a patient with CRC and its isogenic mutant 11G5ΔclbQ, depleted for the *clbQ* gene in the *pks* island and unable to produce colibactin [21] were grown at 37 °C in Luria-Bertani medium overnight.

### 4.2. Animal Model and Infection of Mice

Mice deficient for *Atg16l1* specifically in IECs (*Atg16l1^flox/flox^CreVillin* mice, hereafter termed *Atg16l1^ΔIEC^*) and their littermates *Atg16l1^flox/flox^* were housed in specific pathogen-free conditions. Mice were uninfected or infected with the 11G5 strain or the mutant 11G5ΔclbQ as previously described [21]. Briefly, mice were given streptomycin (2.5 g/L) dissolved in drinking water during 3 days, and then received regular water during 24 h before infection. Each mouse was orally administrated by gavage with 10^9^ bacteria in 200 µl of PBS or with 200 µL of PBS alone (+PBS or uninfected condition). Seven days later, mice were subjected to 12 cycles of DSS treatment, with each cycle consisted of 1% (*w*/*v*) of DSS (36–50 kDa; MP Biomedicals, Irvine, CA, USA) in drinking water for 5 days followed by 14-day of regular water to allow the recovery.

After 12 cycles of DSS treatment, mice were sacrificed. The colon was cut lengthwise, one part was swiss-rolled, fixed in buffered 10% formalin and embedded in paraffin, and another part was frozen at −80 °C for protein extraction.

### 4.3. Ethical Statement

Mice were housed in the specific pathogen-free animal facility at the University of Clermont Auvergne. Mice were fed standard chow ad libitum, had free access to sterile water, and were subjected to 12 h light/12 h dark cycles. Animal protocols were in accordance with the recommendations of the Guide for the Care and Use of Laboratory Animals of the University of Clermont Auvergne and were approved by the French Ministry of National Education, Higher Education and Research (APAFIS#11254).

### 4.4. Clinical Activity Score

Clinical activity score as determined based on assessment of body weight loss, stool consistency, and the presence of occult/gross blood in the stool by a guaiac test (Hemoccult Sensa; Beckman Coulter, Brea, CA, USA) after each DSS treatment for each mouse. Body weight change was scored as follows: 0, no change; 1, 1–5% weight loss; 2, 5–10% weight loss; 3, 10–18% weight loss; 4, >18% weight loss. Stool character was scored as follows: 0, normal, well-formed pellets; 1, soft without pellets; 2, diarrhea. Occult blood was scored as follows: 0, no blood; 1, positive hemoccult (Beckman Coulter); 2, gross bleeding. These scores were added to generate a clinical activity score ranging from 0 to 8.

### 4.5. Hematoxylin and Eosine Staining and Histological Examination

Mouse colons were embedded in paraffin and cut into 5-μm sections with a microtome, and colonic sections were H&E-stained. The histological evaluation (inflammation score and neoplasia score) of the colonic sections was performed under blinded conditions by an expert pathologist according to the criteria described in Table 1 and Table 2.

### 4.6. Immunohistochemical Staining

The sections were deparaffined in Histoclear for 15 min (2 times), rehydrated in ethanol diminishing gradient (100%, 96%, 75% and water: 1 min each) and unmasked in Tris-EDTA buffer (10 mM Tris-Base, 1 mM EDTA, pH 9) during 20 min at 95 °C. The sections were incubated with blocking buffer (1% bovin serum albumin in PBS) for 1 h at room temperature and then with anti-phospho-H2AX (dilution 1/500, #9718, Cell Signaling, Danvers, MA, USA) overnight at 4 °C. After several washes with PBS, the sections were incubated with the corresponding secondary antibody coupled with peroxydase (dilution 1/500, #111-065-003, Jackson Immunoresearch, Ely, Cambridgeshire, United Kingdom) for 2 h at room temperature. Revelation was performed using 3,3′-Diaminobenzidine (#SK-4800, NovaRED, Vector Laboratories, Burlingame, CA, USA). The sections were counterstained with Mayer hematoxilin (Diapath, Martinengo (BG) Italy) for 10 sec, rinsed under running water, dehydrated and mounted in Eukitt mounting solution. The microscopic images were acquired using the Scanner Zeiss Axioscan Z1 (Zeiss, Jena, Germany) and analysed using ZEN 2 software.

### 4.7. Protein Extraction and Western Blot

Extraction of proteins from mouse colonic mucosa and Western blot analysis were performed as previously described [40,41]. The primary antibodies used were anti-LC3 (#L8918, Sigma-Aldrich, Saint-Louis, MO, USA), anti-phospho-H2AX (#2577, Cell Signaling) and anti-α-tubulin (#2144, Cell Signaling). The secondary antibody used was HRP-conjugated anti-rabbit (#7074, Cell Signaling). Blots were detected using the Enhanced Chemiluminescence Detection kit (RPN2108, Amersham Biosciences, Buckinghamshire, UK) and revealed using the ChemiDocTM XRS System (BioRad, Hercules, CA, USA).

### 4.8. Statistical Analysis

Statistical analyses between 2 or several groups were performed using the Student *t* test (Mann–Whitney if not parametric) or analysis of variance (ANOVA) followed by a post-test Bonferroni correction (Kruskal–Wallis if not parametric), respectively, with GraphPad Prism 5 (GraphPad Software, San Diego, CA, USA). A *P* value less than 0.05 was considered as significant.

## 5. Conclusions

The current study showed that colibactin-producing *E. coli* induce the formation of invasive carcinomas in a chronic inflammation-associated mouse model that lacks genetic susceptibility or the use of a carcinogen, and this is further enhanced by autophagy deficiency in intestinal epithelial cells. Thus, our study importantly highlights (i) the role of CoPEC in the initiation of colorectal tumorigenesis, and (ii) autophagy as a key mechanism of host defense against the carcinogenic properties of CoPEC.

## Figures and Tables

**Table 1:** Neoplasia score. To determine the neoplasia score, the criteria in the following table were examined for each mouse, giving a score. This score was then multiplied by “1” if 0–50% of the tumors of the mouse were invasive carcinomas and “2” if 50–100% of the tumors of the mouse were invasive carcinomas. The final value is the neoplasia score.

Evaluation Criteria	Score
No cancer: Normal gland architecture	0
Low-grade dysplasia	1
Moderate dysplasia	2
High-grade dysplasia, carcinoma in situ	3
Invasive carcinoma	4

**Table 2:** Inflammation score.

Evaluation Criteria	Score
Crypt hyperplasia	0: Absent
	1: Mild
	2: Moderate
	3: Marked
Crypt architecture	0: Intact crypt
	1: Irregular crypt (non-parallel crypts, variable crypt diameters, bifurcation and branched crypts)
	2: Crypt loss
Ulceration	0: Absent
	1: Present
Loss of surface epithelium	0: Absent
	1: Focal
	2: Extended
Abscess	0: Absent
	1: Focal
	2: Extended
*Lamina propria* inflammation	0: No inflammation or rare inflammatory cells in the *lamina propria*
	1: Minimal, focal (increased inflammatory cells in the *lamina propria*)
	2: Moderate, mild extended (inflammatory cells extending into the submucosa)
	3: Transmural extension of the inflammatory infiltrate
Sub mucosa inflammation/edema	0: Absent
	1: Slight
	2: Moderate
	3: Severe

## Data Availability

The data presented in this study are available on request from the corresponding author.

## Supplementary Materials

The following are available online at https://www.mdpi.com/article/10.3390/cancers13092060/s1, Figure S1: The uncropped Western blots.

## Abbreviations

*Atg16l1^∆IEC^* mice: mice deficient for *Atg16l1* specifically in intestinal epithelial cells; AOM: azoxymethane; CoPEC: colibactin-producing *Escherichia coli*; CRC: colorectal cancer; DSB: double-strand break; DSS: dextran sodium sulfate; IECs: intestinal epithelial cells; IL: interleukin; PBS: phosphate-buffered saline; pks: polyketide synthase; TNM: tumor-node-metastasis.
