# Supplementary material for: Colibactin-Producing Escherichia coli Induce the Formation of Invasive Carcinomas in a Chronic Inflammation-Associated Mouse Model
Source: Cancers (Basel). 2021 Apr 24;13(9):2060. doi: 10.3390/cancers13092060 (PMC8123153; doi:10.3390/cancers13092060)
Supplement: Supplementary file 1 [file cancers-13-02060-s001.zip › cancers-1200085-supplementary.pdf]

cancers

MDPI

Supplementary Materials: Colibactin-Producing *Escherichia coli* Induce the Formation of Invasive Carcinomas in a Chronic Inflammation-Associated Mouse Model

Laurène Salesse, Cécily Lucas, My Hanh Thi Hoang, Pierre Sauvanet, Alexandra Rezard, Philip Rosenstiel, Christelle Damonsoubeyrand, Nicolas Barnich, Catherine Godfraind, Guillaume Dalmasso and Hang Thi Thu Nguyen

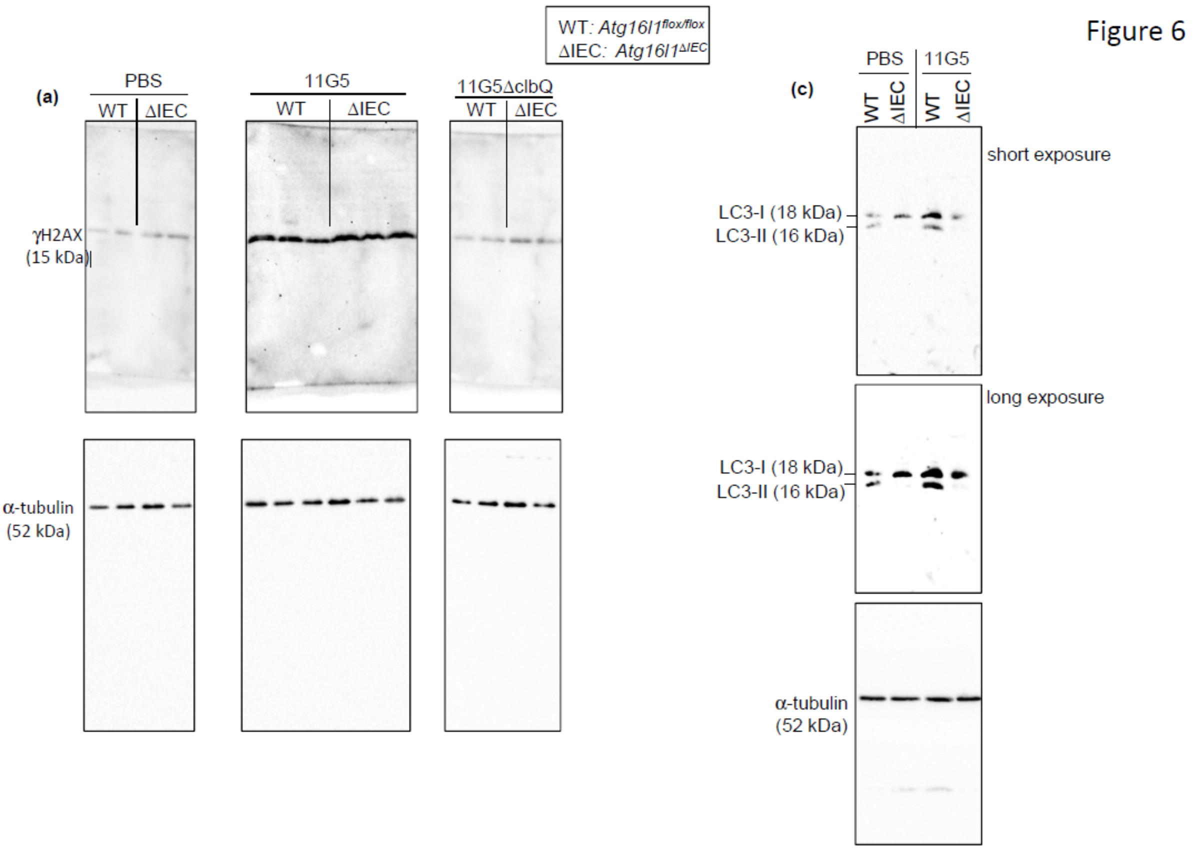

Figure S1. The uncropped Western blots.
